# Sphingolipids modulate the epithelial–mesenchymal transition in cancer

**DOI:** 10.1038/cddiscovery.2015.1

**Published:** 2015-10-12

**Authors:** T Levade, N Andrieu-Abadie, O Micheau, P Legembre, B Ségui

**Affiliations:** 1 INSERM UMR 1037, Centre de Recherches en Cancérologie de Toulouse (CRCT), Oncopole de Toulouse, Toulouse, France; 2 Equipe Labellisée Ligue Contre Le Cancer, Toulouse, France; 3 Université Toulouse III - Paul Sabatier, Toulouse, France; 4 INSERM UMR866, 7 Boulevard Jeanne d'Arc, Dijon, France; 5 Université de Rennes-1, CLCC Eugene Marquis, ER440-OSS, Label INSERM, Rue de la Bataille Flandres Dunkerque, Rennes, France; 6 Equipe Labellisée Ligue Contre Le Cancer, Rennes, France

The epithelial–mesenchymal transition (EMT) allows epithelial cells to acquire biochemical and biological features of mesenchymal cells, increasing cell motility and invasiveness properties, as well as resistance to cell death. EMT is involved not only in embryogenesis and tissue regeneration but also in cancer progression.^1^ Sphingolipids (SL) are sphingoid base-containing lipids, which are structural components of membranes and behave as bioactive molecules in various pathophysiological contexts including cancer.^2^ A recent literature indicates that EMT is likely associated with changes in SL metabolism, and some SL metabolites likely modulate EMT. As a matter of fact, whereas EMT affects the expression of genes encoding SL-metabolizing enzymes and, consequently, alters the sphingolipidome, pharmacological or (epi)genetic modulation of SL metabolism impairs some EMT features (Figure 1).

The first evidence indicating that EMT triggers SL metabolism changes was provided by Hakomori's group, showing that transforming growth factor-*β* (TGF*β*), a well-known EMT inducer,^1^ alters ganglioside (i.e., sialic acid-containing glycosphingolipids) metabolism in normal mouse mammary gland (NMuMG) and bladder cancer (HCV29) epithelial cell lines.^3^ TGF*β*-induced EMT was accompanied by a reduction of Gg4 (asialo-GM1 also known as GA1) and GM2, which belong to the 0- and a-series gangliosides, respectively.^3^ More recently, TGF*β* treatment of NM18 cells, which derive from NMuMG, was shown to increase the intracellular content of LacCer, the ganglioside precursor, as well as a-series gangliosides (GM3, GM2 and GM1a).^4^ Overexpressing the EMT transcription factor inducers Twist or Snail in H-Ras-transformed human mammary epithelial cells (HMLER) increases the level of the b-series ganglioside GD2,^5^ further arguing that EMT is associated with the alteration of ganglioside metabolism.

The modulation of ganglioside pattern upon EMT is likely related, at least in part, with the alteration of gene expression encoding ganglioside metabolism enzymes. TGF*β* and hypoxia-induced EMT of NMuMG cells are associated with a significant reduction of UDP-Gal:β1,3-galactosyltransferase-4 (*β*3GalT4) mRNA encoding Gg4 synthase (Gg4S).^6^ Conversely, the expression of GM3 synthase (GM3S) and GD3 synthase (GD3S) is increased upon TGF*β* treatment of NM18 cells and human breast epithelial cell MCF10A, respectively.^4,7^ Of note, GM3S and GD3S are critical enzymes for the synthesis of a-series (such as GM3, GM2 and GM1a) and b-series (such as GD2) gangliosides, respectively. Different transcription factors involved in EMT have been recently identified to regulate the expression of genes encoding ganglioside-metabolizing enzymes. The Smad3/4 complex binds to and inhibits the β3GalT4 promoter.^8^ Zeb1 (zinc-finger E-box binding homeobox 1) binds to and activates the promoters of both GM3S^4^ and GD3S^9^ genes. Moreover, Zeb1 impairs the expression of miR-200a, a microRNA targeting the 3ʹUTR GM3S mRNA.^4^ Overexpressing Twist or Snail in HMLER cells enhances the expression of GD3S.^5^ Moreover, NF-*κ*B-dependent FOXC2 activation triggers GD3S expression in both human triple-negative breast cancer (TNBC) MDA-MB-231 and Snail-overexpressing HMLER cells.^7^

A growing body of evidence indicates that the modulation of glycosphingolipid metabolism has a critical role in EMT. Pharmacological inhibition of the glucosylceramide synthase, which drives the synthesis of most glycosphingolipids, triggers EMT in diverse epithelial cells.^3^ Treatment with exogenous Gg4 or Gg4S overexpression prevents TGF*β-*triggered EMT of NMuMG cells.^3,6^ Gg4 was reported to physically interact with key epithelial cell molecules, such as E-cadherin and *β*-catenin, likely facilitating epithelial intercellular adhesion via stabilization of the E-cadherin/*β*-catenin complex at the cell surface.^6^ However, conflicting observations, have been reported concerning the role of GM3S in EMT. Overexpression of GM3S in A2780 human ovarian carcinoma cell lines decreases the expression of the EMT marker *α*-smooth muscle actin and impaired cell motility.^10^ Opposite findings were described in mouse NM18 cells in which the knockdown of GM3S increases the expression of adhesion molecules such as E-cadherin and, consequently, intercellular adhesion, whereas the GM3S overexpression has the opposite effect.^4^ Regarding GD3S, its knockdown in mesenchymal cell lines (i.e., HMLER and MDA-MB-231) triggers the expression of E-cadherin and the concomitant downregulation of N-cadherin and vimentin mesenchymal markers (Figure 1). GD3S silencing also reduces the motility of breast cancer cells *in vitro* and metastasis in mice via inhibition of the hepatocyte growth factor/c-Met signaling pathway. Interestingly, meta-analysis of GD3S expression indicates that GD3S is upregulated in TNBC, which exhibit a mesenchymal-like gene signature, and high expression of GD3S is associated with a bad prognosis.^7^

EMT also affects other SL metabolites than gangliosides, as illustrated in Mardin–Darby canine kidney cells undergoing EMT upon expression of a constitutively active Raf1 mutant and TGF*β* treatment. Whereas sphingomyelin level significantly increases when cells become mesenchymal, sulfatides become almost undetectable.^11^ Incubation of the human prostate cancer line DU145 with endocrine disruptors triggers EMT along with a reduction of the intracellular content of dihydroceramide, ceramide, glycosphingolipids and sphingomyelin.^12^ We have recently documented that EMT modulates the expression of ceramide synthase 6 (CerS6),^13^ which preferentially synthesizes SLs with a C16:0 long-chain saturated fatty acid.^14^ TGF*β* triggers downregulation of CerS6 mRNA expression in human breast epithelial cells, and CerS6 mRNA and protein levels are reduced in mesenchymal-like cells, including TNBC as compared with epithelial-like cells such as non-TNBC. Of note, changes in CerS6 expression in human breast cancer cells do not alter the expression of the epithelial (E-cadherin) and mesenchymal (vimentin) markers, indicating that CerS6 *per se* does not control EMT. CerS6 preferentially produces C16:0 dihydroceramide, which is further metabolized to C16:0 ceramide and more complex C16:0 SLs, such as C16:0 sphingomyelin and glycosphingolipids, which are both enriched in the plasma membrane microdomains.^2^ Our study pinpoints that the downregulation of CerS6 in TNBC is associated with an increased plasma membrane fluidity, which favors breast cancer cell motility. Whereas CerS6 knockdown in non-TNBC cell lines elicits a decrease in C16:0 ceramide intracellular content and an increased membrane fluidity, its overexpression in TNBC as well as exogenous C16:0 ceramide have opposite effects. Consequently, CerS6 overexpression and exogenous C16:0 ceramide impair breast cancer cell motility triggered by a non-apoptotic and pro-motile metalloprotease-cleaved CD95L.^13^ The soluble form of CD95L is produced at high levels in patients affected with TNBC and triggers the formation of a motility-inducing signaling complex.^15^ The low expression of CerS6, associated with a high production of the soluble CD95L in patients affected with TNBC, which exhibit a mesenchymal-like phenotype, may facilitate cancer progression and metastasis. Increasing CerS6 expression and/or C16:0 SL intracellular content and/or preventing the implementation of the CD95 signaling pathway may thus reduce TNBC metastatic dissemination (Figure 1).

Various SL metabolism alterations, which have been reported in cancer cells undergoing EMT, likely modulate cancer cell motility and metastasis. Deciphering the relationships between SL metabolism and EMT may open new therapeutic avenues to limit cancer progression.

## Figures and Tables

**Figure 1:**
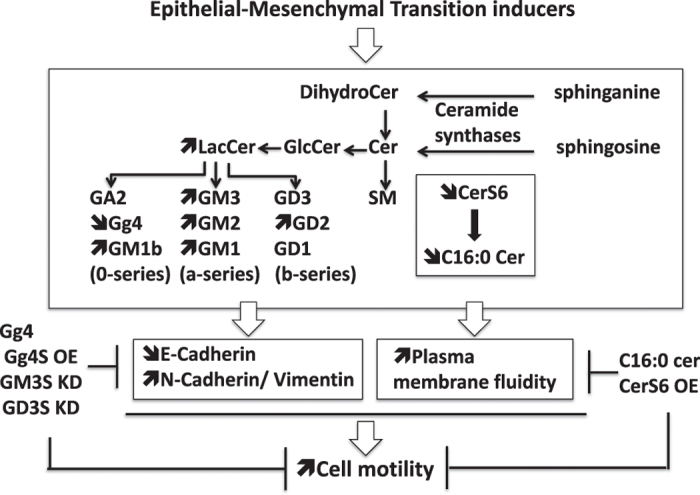
EMT and SL metabolism relationship. EMT alters the expression of SL-metabolizing enzymes such as Gg4S, GM3S, GD3S and CerS6S associated with intracellular SL content variations. SL metabolism alterations modulate EMT features such as E-cadherin downregulation, plasma membrane fluidity increase and cell motility. Exogenous Gg4 and C16:0 ceramide (C16:0 cer) as well as the overexpression of Gg4S (Gg4S OE) and CerS6 (CerS6 OE) inhibit cell motility, as GM3S (GM3S KD) and GD3S (GD3S KD) knockdown does.
